# Quantile regression analysis of the association between parental rearing and interpersonal sensitivity in Chinese adolescents

**DOI:** 10.1186/s12889-021-12487-w

**Published:** 2022-01-11

**Authors:** Yafeng Zhang, Wei Tian, Yuqi Xin, Quan Zhou, Guangcan Yan, Jianqiu Zhou, Bin Wang, Yuchun Tao, Lihua Fan, Limin Wang

**Affiliations:** 1 Department of Health Management, School of Health Management, Harbin Medical University, No.157 Baojian Road, Harbin, 150081 China; 2 Department of Biostatistics, School of Public Health, Harbin Medical University, No.157 Baojian Road, Harbin, 150081 China; 3 Department of Health Education, School of Health Management, Harbin Medical University, No.157 Baojian Road, Harbin, 150081 China; 4 Centre for Experimental Teaching of Functional Sciences, School of Basic Medicine, Harbin Medical University, No.157 Baojian Road, Harbin, 150081 China; 5 Nanbai High School of Zunyi City, Guihua Community, Longkeng Town, Bozhou District, Zunyi, 563100 China

**Keywords:** Parenting rearing, Interpersonal sensitivity, Quantile regression, Family education

## Abstract

**Background:**

Parental rearing is well documented as an important influencing factor of interpersonal sensitivity (IS). However, little research has focused on the extent by which various aspects of parental rearing in fluence IS. This study aimed to analyze the effects of parental rearing on IS, using quantile regression. We analyzed the extent of the influence of parental rearing on IS by quantile regression to provide definitive evidence on the family education of adolescents with IS problems.

**Methods:**

The multiple cross-sectional studies were conducted among 3345 adolescents from Harbin, China, in 1999, 2006, 2009 and 2016. Furthermore, a multistage sampling method (stratified random cluster) was used to select participants. IS was assessed using a subscale of the Symptom Checklist-90-Revision. Perceived parental rearing was assessed using the Egna Minnen av. Barndoms Uppfostran. The ordinary least squares (OLS) linear regression was used to determine the average effect of parental rearing on IS. The quantile regression was conducted to examine the established associations and to further explain the association.

**Results:**

Paternal emotional warmth was found to be associated with IS across the quantile, especially after the 0.6 quantiles; however, this association was not found for maternal emotional warmth. Paternal punishment was associated with IS at the 0.22–0.27 and 0.60 quantile; however, maternal punishment had no significant effect on IS. QR method found that paternal overinvolvement was associated with IS at the 0.48–0.65 quantiles, but paternal overprotection was associated with IS across the quantile; however, maternal overinvolvement and overprotection was positively correlated with IS at the 0.07–0.95 quantiles. The correlation between paternal rejection and IS was found at the 0.40–0.75 and > 0.90 quantiles; maternal rejection was associated with IS within the 0.05–0.92 quantiles.

**Conclusions:**

Parental rearing practices predict different magnitudes of IS at varying levels. This study provides suggestions for parents to assess purposefully and systematically, intervene, and ameliorate adolescent IS problems. We also highlight the role of paternal rearing in children’s IS problems, providing new ideas for family education.

## Background

Interpersonal sensitivity (IS) is a psychological trait defined by an individual’s hypersensitivity to a sense of lack of self in interpersonal interactions [1, 2]. Individuals with IS are preoccupied with interpersonal relationships, vigilant to the behavior and mood of others, sensitive to perceived or actual criticism or rejection, and modify their behavior to comply with others’ expectations [3]. Gillespie used a twin study design to examine the origins of IS and found that IS is also influenced by family environmental factors [4]. Among the family factors, parental rearing is a key variable in the healthy development of children. Parental rearing can be understood as attitudes conveyed to the child, usually conceptualized in terms of two dimensions: parental demand (e.g., control) and parental response (e.g., warmth) [5]. Parental rearing has a lasting impact on children’s well-being because they underlie children’s internalized views of relationships and their general expectations of whether they will be accepted, supported, or rejected by others [6, 7]. There is therefore a close link between parental rearing and IS.

Research suggests that exposure to dysfunctional parental rearing in childhood may increase susceptibility to mental disorders such as IS [8–10]. One study found that in male subjects, higher IS scores were related to higher scores of paternal protection and maternal protection, while in female subjects, higher IS scores were related to higher scores of maternal protection [10]. Studies have also shown that the interaction between parental rearing, especially maternal care, and the brain-derived neurotrophic factor (BDNF) Val66Met polymorphism affects interpersonal sensitivity in healthy subjects [11]. However, although research has confirmed the correlation between parental rearing and IS, few studies have reported the extent to which parental rearing is associated with IS. Moreover, evidence on whether and how the associations would vary according to the quantiles of IS and the type of parental rearing is limited. Therefore, in order to measure the range of correlations between IS and parental rearing, this study used quantile regression (QR) methods to re-examine the established associations between parental rearing and IS and to further explain the association.

QR analysis was introduced by Koenker and Bassett in 1978 as a modelling approach for the association between one or more explanatory variables and continuous outcome variables [12]. The QR model has the advantage of being much more robust to outliers than ordinary least squares regression, avoiding the assumption of parameter distributions in the error process and being a powerful tool for estimating the conditional distribution of outcomes [13]. In the medical field, the use of QR models has been focused on the anesthesia and health economics research [14, 15]. As a valid tool for correlational research, there has been a gradual increase in the field of psychiatry (e.g. suicidal ideation and obsessive-compulsive disorder) in recent years [16, 17]. However, few studies have used the QR approach to IS. The QR coefficient estimates are more robust, and the QR approach provides more information on the data to obtain the effect of parental rearing on IS at each quantile, which can provide concrete evidence that family education improves children’s level of IS.

The purpose of the present study was to determine the possible differences in associations of parental rearing across the quantile levels of IS, using QR approach. Consequently, the present study reexamined established correlates between parental rearing and IS, using the four cross-sectional data from decades. This study hypothesized that parental rearing practices act as predictors of different magnitudes at varying levels of IS.

## Methods

### Participants and procedures

The findings presented here were drawn from multiple cross-sectional studies conducted in Harbin, China, on adolescent IS in 1999, 2006, 2009, and 2016. Four schools were selected from a region in Harbin, China, using a multistage sampling method (stratified random cluster) [18]. Among the four schools, two were key schools and two were common schools. The second stage was a whole-group sampling by grade level. Two classes were selected as units from each grade (grades 7, 8, 10 and 11) in the selected school, and the respondents were selected. These students range in age from 12 to 20 years and are in transition from the role of a child to that of an adult. These four surveys were conducted according to the survey procedures described above.

The WHO summary of the literature shows that mental disorders are prevalent in approximately 20% of children and adolescents in developed countries and a few developing countries [19, 20]. Based on the sample size formula for cross-sectional surveys**(**$\boldsymbol{N}={\boldsymbol{\mu}}_{\frac{\boldsymbol{\alpha}}{\mathbf{2}}}^{\mathbf{2}}\boldsymbol{\pi} \left(\mathbf{1}-\boldsymbol{\pi} \right)/{\boldsymbol{\delta}}^{\mathbf{2}}$**)**, a sample of about 683 participants was measured with an error tolerance of no more than 3%. To avoid invalid questionnaires, we have determined a sample size of approximately 800 participants for each survey. A total of 3441 questionnaires were therefore distributed at the four survey time points.

**Fig. 1:**
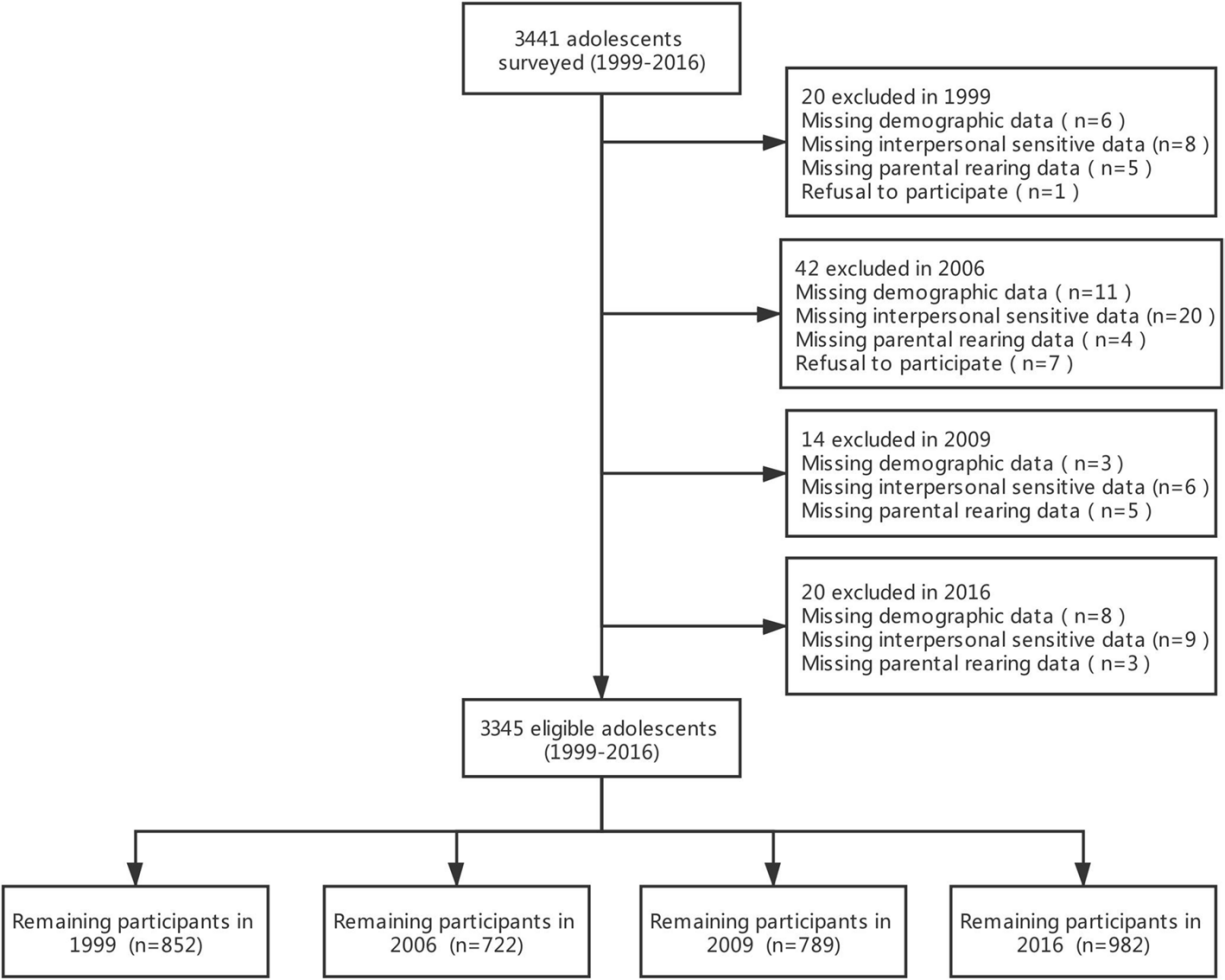
Samples flow chart

After excluding 96 students, 3345 students were surveyed, with a response rate of 97.21% (Fig. 1). The baseline survey was conducted in 1999 (*N* = 852; 25.5%), and the follow-up surveys were conducted in 2006 (*N* = 722; 21.6%), 2009 (*N* = 789; 23.6%), and 2016 (*N* = 982; 29.4%). Among the respondents, 1243 (37.2%) were only children, 2102 (62.8%) were non-only children, 224 (6.7%) were from single-parent families, 448 (13.4%) had a strained parental relationship, and 323 (9.7%) had parents who often quarreled. As the sampling method chosen was stratified random cluster (class as a cluster), the inclusion criteria for this study were students who were in the class at the same time during the survey and who answered the questionnaire information completely. The exclusion criteria were no demographic information (*N* = 28) or incomplete IS items (*N* = 43) and EMBU scale (*N* = 17). Adolescents who refused to participate (*N* = 8) were also excluded. Grade 9 and 12 students were excluded because they were preparing for entrance examinations.

Trained researchers explained the purpose of the study to each school and the students, and the research was conducted by completing an anonymous, structured, self-report questionnaire that they completed in the classroom. The complex questionnaire items were further explained in the instructions. The average time spent per survey was approximately 40 min. All participants were informed that participation was voluntary and that their responses would be confidential. This study was approved by the Ethics Committee of Harbin Medical University (Study: ID HMUIRB20210003) and was conducted according to the tenets of the 1964 Helsinki Declaration and its later amendments.

### Measures

*Symptom Checklist-90-Revision (SCL-90-R)*. The SCL-90-R is a 90-item self-report measurement that includes questions on the following psychological symptoms: somatization (12 items), obsessive-compulsive (10 items), IS (9 items), depression (13 items), anxiety (10 items), hostility (6 items), phobic anxiety (7 items), paranoid ideation (6 items), and psychoticism (10 items). The nine-item IS subscale was extracted from the SCL-90-R [21]. The nine items included feeling critical of others, feeling shy of opposite sex, feeling easily hurt, others are unsympathetic, people dislike you, feeling inferior to others, uneasy when people are watching you, self-conscious with others, and uncomfortable eating/drinking in public. Each item of the questionnaire is rated on a 5-point scale ranging from 0 (not at all) to 4 (very much), with higher scores indicating higher levels of IS [22, 23].

The mean score of the IS factor (i.e., total IS scale score/number of items) was used in this study. The SCL-90-R assesses the respondents’ feelings during the last 2 weeks. The scale is widely used among Chinese students and has good validity and reliability [24]. Further, the SCL-90-R has been demonstrated to be psychometrically reliable [25]. The SCL-90-R demonstrated strong psychometric properties and excellent internal consistency in the present study (Cronbach’s coefficient alpha = 0.96). The Cronbach’s alpha coefficient of the IS subscale was 0.86. With respect to construct validity, a high factor loading (> 0.50) was exhibited in the IS subscale according to the exploratory factor analysis.

#### Egna Minnen av. Barndoms Uppfostran (EMBU)

The EMBU is a 66-item self-report scale that assesses one’s memory of parental rearing and consists of four replicate dimensions: rejection, emotional warmth, overprotection, and favoritism. These are reduced from 11 aspects of parental behavior and attitudes: father rearing (emotional warmth, F1; punishment, F2; overinvolvement, F3; favoritism, F4; rejection, F5; overprotection, F6) and mother rearing (emotional warmth, M1; overinvolvement and overprotection, M2; rejection, M3; punishment, M4; favoritism, M5) [26]. Meanwhile, there were significant differences in parental rearing between Chinese parents (e.g., in paternal rearing, overinvolvement (F3) and overprotection (F6) are two independent predictors, while maternal overinvolvement and overprotection (M2) is an independent predictor). The parental preference factors refer to the fact that my parents prefer one sibling over another siblings. Given China’s “one-child” policy from 1980 to 2015, many families have only one child. Considering the missing data, we removed the paternal favoritism (F4) and maternal favoritism (M5) factors.

Responses were rated on a 4-point scale from 1 (never) to 4 (always). Each parental rearing practice was obtained by adding the corresponding item scores, with higher scores indicating stronger memories of the corresponding parental rearing. The EMBU was found to have good internal reliability and consistency coefficients for parental rearing by fathers and mothers (0.87 and 0.79, respectively). With respect to construct validity, high factor loadings for parental rearing by fathers and mothers were found (> 0.77 and > 0.86, respectively). The alpha coefficients for fathers’ emotional warmth, punishment, overinvolvement, rejection, and overprotection were 0.87, 0.87, 0.73, 0.73, and 0.76, respectively. For the mothers, the alpha coefficients for emotional warmth, overinvolvement, overprotection, rejection, and punishment were 0.91, 0.75, 0.82, and 0.85, respectively. The Chinese EMBU version has been shown to have high reliability and validity [27].

### Statistical analysis

Correlation between rank variables was measured using Spearman rank correlation coefficients. Linearity and closeness between quantitative variables were measured using Pearson correlations. The significance of correlation of variables was checked using a t-test. *P*-values after Bonferroni correction were used. Continuous data are presented as the mean ± standard deviation (SD). Previous studies showed that age and sex may have an impact on IS [11, 28]. In addition, to avoid the influence of different survey years on the development of IS, we used sex, grade, and survey years as control variables in the data analysis.

Then, ordinary least squares (OLS) linear regression was used to determine the average effect of each predictor on IS. Finally, we applied a QR analytical approach to evaluate the association between parental rearing and IS with a set of quantile ranges of 0.05–0.95. The quantile regression (QR) model can measure particularly weaker or more potent correlates between parental rearing and IS at different levels. Compared with linear regression, QR extends to testing the effect of a predictor variable on an outcome variable at varying levels of the outcome variable rather than presuming a uniform mean effect [16].

In addition, the differential associations might be overlooked when using typical mean regression models, such as ordinary least-squares regression, but could be captured using a QR model [29]. Based on the regression coefficients for the association between IS levels and parental rearing derived from the QR model, we created a visualization of the correlation between parental rearing and IS. The graph shows the change in IS levels at one unit of parental rearing at the quantile level of IS. All statistical analyses were performed using SAS software (version 9.3; SAS Institute Inc., Cary, NC), and statistical significance was set at *p* <  0.05.

## Results

The correlations between the SCL-90 internal subscale and IS subscale items were statistically significant (Table 1), with correlations exceeding 0.50 for the SCL-90 internal subscale and being between 0.23 and 0.64 for the IS subscale items. The bivariate correlations and descriptive statistics of parental rearing and IS are presented in Table 2. Except for the insignificant correlation between the F1 and M2 variables, all other variables were significantly associated with each other. We compared the correlations between the same paternal and maternal rearing variables. As expected, there was a significant positive correlation between F1 and M1, with a correlation coefficient of 0.75. F2 and M4 showed a significant positive correlation (correlation coefficient is 0.60); F3 and M2 had a significant positive correlation (correlation coefficient is 0.58); F5 and M3 had a significant positive correlation (correlation coefficient is 0.61); and F6 and M2 had a significant positive correlation (correlation coefficient is 0.52). Table 2 also shows that the mean IS score was 1.99, SD = 0.73.

**Table 1:** Correlations between SCL-90 internal subscales and between items within the IS subscale (*n* = 3345)

	SOM	O-C	I-S	DEP	ANX	HOS	PHOB	PAR	PSY	Other
SOM	1									
O-C	0.65^a^	1								
I-S	0.6^a^	0.76^a^	1							
DEP	0.67^a^	0.77^a^	0.83^a^	1						
ANX	0.75^a^	0.76^a^	0.77^a^	0.82^a^	1					
HOS	0.6^a^	0.65^a^	0.69^a^	0.69^a^	0.71^a^	1				
PHOB	0.56^a^	0.59^a^	0.64^a^	0.63^a^	0.7^a^	0.52^a^	1			
PAR	0.61^a^	0.72^a^	0.8^a^	0.76^a^	0.75^a^	0.69^a^	0.58^a^	1		
PSY	0.68^a^	0.75^a^	0.78^a^	0.81^a^	0.81^a^	0.68^a^	0.62^a^	0.77^a^	1	
Other	0.65^a^	0.67^a^	0.66^a^	0.70^a^	0.71^a^	0.63^a^	0.54^a^	0.63^a^	0.71^a^	1
The correlation between the items (number) in the IS subscale										
Feeling critical of others	1									
Feeling shy opposite sex	0.26^b^	1								
Feeling easily hurt	0.31^b^	0.37^b^	1							
Others are unsympathetic	0.33^b^	0.33^b^	0.49^b^	1						
People dislike you	0.33^b^	0.31^b^	0.48^b^	0.64^b^	1					
Feeling inferior to others	0.28^b^	0.29^b^	0.48^b^	0.47^b^	0.51^b^	1				
Uneasy when people are watching you	0.32^b^	0.33^b^	0.51^b^	0.48^b^	0.45^b^	0.53^b^	1			
Self-conscious with others	0.35^b^	0.32^b^	0.52^b^	0.51^b^	0.53^b^	0.48^b^	0.52^b^	1		
Uncomfortable eating/drinking in public	0.23^b^	0.25^b^	0.32^b^	0.33^b^	0.35^b^	0.34^b^	0.38^b^	0.42^b^	1	

Note. SOM: somatization; O-C: obsessive-compulsive; I-S: interpersonal sensitivity; DEP: depression; ANX: anxiety; HOS: hostility; PHOB: phobic anxiety; PAR: paranoid ideation; PSY: psychoticism

^a^ *P* value < 0.00111 was regarded as significant after Bonferroni corrected

^b^ *P* value < 0.00138 was regarded as significant after Bonferroni corrected

Correlations (r) between subscales within the SCL-90 were measured using Pearson correlations, and internal correlations for the IS subscales were measured using Spearman rank correlation coefficients, both of which were checked for significance using t-tests

**Table 2:** Bivariate Correlations and Descriptive Statistics of parental rearing and IS (n = 3345)

Variables	1	2	3	4	5	6	7	8	9	10
1.F1	1									
2.F2	−0.11^c^	1								
3.F3	0.17^c^	0.54^c^	1							
4.F5	−0.08^c^	0.68^c^	0.56^c^	1						
5.F6	0.24^c^	0.43^c^	0.58^c^	0.49^c^	1					
6.M1	0.75^c^	−0.15^c^	0.05	−0.14^c^	0.10^c^	1				
7.M2	0.03	0.38^c^	0.58^c^	0.42^c^	0.52^c^	0.13^c^	1			
8.M3	-0.22^c^	0.50^c^	0.37^c^	0.61^c^	0.35^c^	-0.20^c^	0.60^c^	1		
9.M4	-0.16^c^	0.60^c^	0.31^c^	0.44^c^	0.26^c^	-0.13^c^	0.53^c^	0.69^c^	1	
10.IS	-0.16^c^	0.26^c^	0.22^c^	0.31^c^	0.24^c^	-0.10^c^	0.32^c^	0.37^c^	0.27^c^	1
Mean	45.95	15.40	18.77	8.47	10.17	50.24	33.48	12.32	12.16	1.99
SD	11.06	5.00	4.60	2.76	2.98	11.21	7.77	4.12	3.82	0.73

Note. Father rearing, emotional warmth = F1; punishment = F2; overinvolvement = F3; rejection = F5; overprotection = F6. Mother rearing, emotional warmth = M1; overinvolvement and overprotection = M2; rejection = M3; punishment = M4. IS = interpersonal sensitivity. SD = standard deviation

^c^ P value < 0.00111 was regarded as significant after Bonferroni corrected

The correlation (r) between parental rearing and IS was measured using Pearson correlation, while significance was also checked using t-tests

We conducted OLS linear regression, examining the mean effect of parental rearing on IS (Table 3). F1 (β = − 0.010; 95% CI − 0.014, − 0.007), F2 (β = 0.011; 95% CI 0.002, 0.021), F5 (β = 0.019; 95% CI 0.001, 0.037), F6 (β = 0.035; 95% CI 0.022, 0.048), M2 (β = 0.013; 95% CI 0.008, 0.019) and M3 (β = 0.039; 95% CI 0.027, 0.051) were correlated with IS in OLS linear regression. F3, M1 and M4 were not correlated with IS in OLS linear regression.

**Table 3:** Marginal associations of IS with parental rearing at the mean levels of IS (*n* = 3345)

Variables	Coefficients	95% CI	*t*	*P* value
F1	−0.010	−0.014, −0.007	−5.86	< 0.001
F2	0.011	0.002, 0.021	2.46	0.014
F3	−0.009	−0.018, 0.001	−1.84	0.066
F5	0.019	0.001, 0.037	2.11	0.035
F6	0.035	0.022, 0.048	5.41	< 0.001
M1	0.001	−0.003, 0.004	0.38	0.707
M2	0.013	0.008, 0.019	4.48	< 0.001
M3	0.039	0.027, 0.051	6.40	< 0.001
M4	−0.01	−0.022, 0.002	−1.60	0.109

Note. 95% CI, 95% confidence intervals. All estimations were adjusted for grade (senior/junior), sex (girls/boys), survey years (2016/other years)

**Fig. 2:**
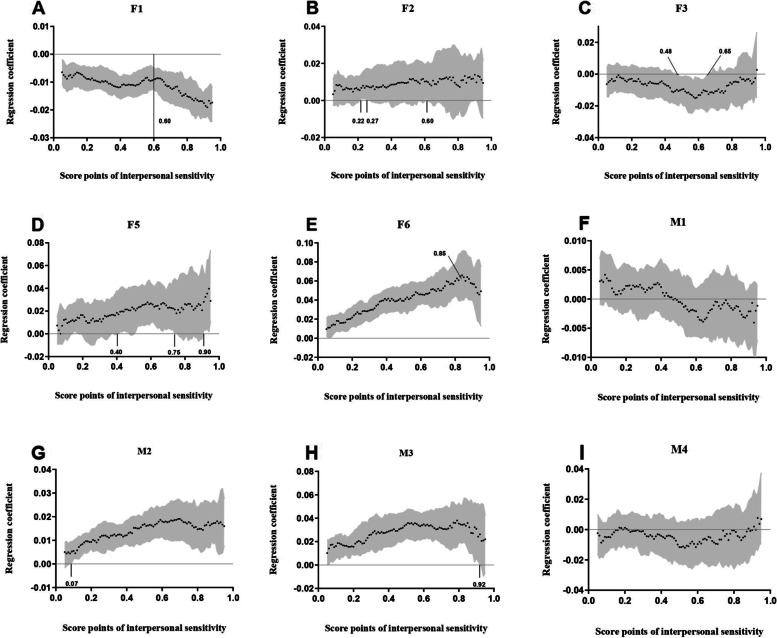
Quantile regressions predicting interpersonal sensitivity at the 0.05–0.95 quantile. Coefficients (β) for the associations of interpersonal sensitivity with parental rearing across 0.05–0.95 quantile. The black solid horizontal line represents β = 0, black dots represent the estimated coefficients and the grey area represents 95%CI of the corresponding parameters. All estimations were adjusted for grades, sex, survey years

Given the difference in the association between parental rearing and IS in the linear regression model and QR model, we conducted a quantile range analysis from 0.05 to 0.95. Figure 2 shows the quantile levels of IS (range, 0.05 to 0.95) on the x-axis and the regression coefficients for the associations of IS quantile levels with parental rearing (β) derived from QR models on the y-axis. The 95% CI of the regression coefficients is also shown in the figure. If the regression coefficient (β) and 95% CI of this quantile crossed 0 on the y-axis, this quantile was not statistically significant.

The association of IS and F1 was found to be significant at each quantile, and the negative correlation was most obvious after the 0.60 quantile. However, M1 was not significantly correlated with IS in the QR model (0.05–0.95 quantiles). In the entire QR analysis, F2 was significant at the 0.22–0.27 and 0.60 quantile. In addition, M4 had no significance for IS at any quantile. Meanwhile, the relationship between F3 and IS was significant at the 0.48–0.65 quantiles. There was also a significance for M2 at the 0.07–0.95 quantiles. The results also showed that F5 had a positive effect on IS at 0.40–0.75 and > 0.90 quantiles. At the 0.05–0.92 quantiles of IS, M3 also had a significant association with IS. F6 was positively correlated with IS at each quantile and was a stronger predictor at the high end of the quantile than at the low end, but the relationship began to decline again at the 0.85 quantile.

## Discussion

The current study found that the effect of paternal rearing (emotional warmth, punishment, overinvolvement, rejection, and overprotection) and maternal rearing (overinvolvement and overprotection, and rejection) differed in magnitude based on IS levels. Paternal overinvolvement and paternal rejection were associated only with IS in a specific quantile range. The correlation between maternal emotional warmth, maternal punishment and IS was not significant. Importantly, we also found that predictors of the same paternal/maternal rearing variables play different roles in adolescent IS. Although, the mechanism for psychology is yet to be investigated, the results have functional significance for improving children’s and families’ well-being.

The results showed that the predictive capability for IS in adolescents differed between paternal and maternal emotional warmth. The reason for this difference may be that fathers and mothers seem to display different styles in their interactions with the child. Consistently, it has been found that in Chinese families, children are more likely to turn to mothers for emotional support, physical needs, and help in dealing with problems of daily life. Thus, the maternal emotional warmth is important for the development of the children’s intrinsic needs, while IS is more of an extrinsic emotional need, which explains the insignificant correlation with both. Paternal warmth may be expressed as providing guidance and assistance to children in learning social skills, acquiring social status, and achieving in academic areas [30]. Compared with mothers, fathers are more involved in children’s training and play essential roles in parenting [31]. Paternal emotional warmth allows children to explore their environments and thus may be linked to the development of feelings of security, confidence, trust, and positive orientation toward others [30]. As a result, fathers are more likely to provide social support and assistance when their children experience IS problems. Moreover, the role of paternal emotional warmth was more significant in the high IS adolescent, also indicating the importance of positive paternal rearing in adolescent IS. Although the results of both the OLS and QR methods indicated that paternal emotional warmth was negatively associated with IS and maternal emotional warmth was not associated with IS, the QR regression further described the range of variation in parental emotional warmth on IS.

While the OLS method found that paternal punishment was associated with IS, the QR model analyses of the association showed that there was an association between parental punishment and IS at only a few quantiles, and no continuous range of associations with IS emerged. This suggests that the correlation may be influenced by outlier points. Both the OLS and QR methods showed no significant association between maternal punishment and IS. A regular association between parental punishment and IS was also not found with respect to the distribution of regression coefficients. Parental punishment during adolescence is known to hurt interpersonal functioning [32, 33]. Our results further support this association. In the Chinese educational context, this parental rearing of parental punishment (“Showing close (relationship) by beating, showing love by scolding”) is considered an expression of parental involvement, care, and love and is generally considered acceptable [34, 35]. Thus, this explains the non-correlation of parental punishment with IS at almost the entire quantile. However, further research is needed to further confirm the reliability of this association.

In parental rearing, the line between overinvolvement and overprotection is very close. Parental overinvolvement refers to overcontrolling and restricting the child’s attempts at autonomy and demands the child’s obedience to parental requests for control [6]. Parental involvement will lower the children’s independence and increase social fearfulness and difficulty in navigating social relationships, resulting in excessive dependence on parents and heightened anxiety about social encounters [7, 36]. Further, parental overprotection will lead the child to feel oppression unconsciously and automatically from others. Attachment theory proposes that parents who fail to provide a secure base and/or encourage exploration make a child anxious, insecure, dependent, or immature and at risk of psychological problems under stress [37]. A previous study demonstrated that high parental protection (assessed using the Parental Bonding Instrument) increases IS (assessed using the Interpersonal Sensitivity Measure) in children [10]. The OLS method found that maternal overinvolvement and overprotection were associated with IS, while the QR approach found that maternal overinvolvement and overprotection increased with each quantile, resulting in greater IS and suggesting that mothers should reduce this parental rearing.

Meanwhile, paternal overinvolvement and overprotection showed a different pattern. The OLS method found that paternal overprotection was associated with IS and paternal overinvolvement was not. QR method found that paternal overprotection was associated with IS, but paternal overinvolvement was associated with IS only to specific quantiles. This suggests that fathers need to reduce overprotective rearing, but increase appropriately involved rearing rather than over-involved rearing.

The OLS method found that both paternal rejection and maternal rejection were associated with IS. However, the QR model confirms that rearing of maternal rejection has a greater impact on IS than has the rearing of paternal rejection. And paternal rejection affect IS only in the special range. Parental rejection includes overt and covert displays of disliking, dismissing, and disapproving of the child and his or her behavior [7]. The literature indicates that non-supportive parental behavior and parental rejection may be linked to more interpersonal problems [38]. Downey et al. found that children who reported higher amounts of perceived parental rejection showed higher expectations of being rejected in social situations 1 year later [39]. For mothers, therefore, there should be an increase in encouraging and acknowledging rearing and an avoidance of rejecting and denying rearing. Fathers also need to reduce rejection rearing in time for moderately IS children and give warmth and rewards at the right time.

There are some limitations to the study. First, the study combined data from multiple time points for analysis and included time points as covariates in the model. However, it is undeniable that other changes due to temporal factors remain uncontrollable. Further longitudinal data tracking is needed in the future to verify the stability of the association. Second, this study used multiple cross-sectional surveys, precluding any causal interpretations. Third, the present study indicated that EMBU is based on adolescents’ perceptions of parental rearing, but not parental reports, which can be influenced by recall bias and social desirability. Finally, given its psychometric properties, the SCL-90-R is only a suitable instrument to assess psychopathological symptomatology during adolescence.

### Implication practice

Results from four cross-sectional studies over the last 20 years show that IS is one of the main psychological problems that affect adolescents, highlighting that adolescents should be offered preventive interventions to improve their personal IS. The specific steps recommended are timely diagnosis of IS, promotion of self-management, increase in family involvement, and provision of continuous support. Given the important role of parents in the psychological development of their children, parents also need to learn how to educate their children and how to play an active role in their upbringing. They should be trained in the community and in schools about home education, and fathers need to be involved more. Family education is important in improving IS for children, and this study confirms “to what extent” parental rearing matter. This provides a direction for the precise management of family education practices. Future mechanisms of “to what extent” still need to be proven.

The findings of this study also have important value for public health. The promotion of mental health is an important topic in public health. By identifying the unique role of parental rearing in adolescent IS, interventions in this phase can have a catalytic effect in directly improving adolescent mental health. In addition, the empirical findings of this study have important public health value in changing parents’ perceptions of parenting and developing good parenting skills to reduce or eliminate poor parenting practices that affect mental health.

## Conclusion

This study shows that maternal and parental rearing have different effects on IS but are correlated. Furthermore, it provides new insights to explain IS with respect to parent-child relationships and suggests that parents should practice positive parental rearing and avoid negative parenting, while focusing on the range of parental rearing and “too far is as bad as not enough.” More importantly, it also confirms the importance of paternal rearing in children’s interpersonal interactions, highlighting the role of positive paternal rearing.

## Data Availability

The raw/processed data required to reproduce these findings cannot be shared at this time as the data also forms part of an ongoing study. Please contact authors for data requests (Limin WANG PhD - email address: wanglimin2008@163.com).

## Abbreviations

EMBU: Egna Minnen av. Barndoms Uppfostran
IS: interpersonal sensitivity
OLS: ordinary least squares
QR: quantile regression
SCL-90-R: Symptom Checklist-90-Revision
